# Serum cholinesterase (ChE) as an emerging biomarker in traumatic spinal cord injury: A retrospective cohort study

**DOI:** 10.1097/MD.0000000000043922

**Published:** 2025-08-22

**Authors:** Yongchang Li, Liangyun Shen

**Affiliations:** a Trauma Intensive Care Unit, Changzhou Second People’s Hospital, Changzhou, China.

**Keywords:** biomarker, diagnosis, serum cholinesterase (ChE), traumatic spinal injury (TSCI)

## Abstract

Traumatic spinal cord injury (TSCI) involves dynamic pathophysiological processes, including primary mechanical damage and secondary inflammatory cascades. Early biomarkers to assess injury severity and predict recovery remain limited. To evaluate serum cholinesterase (ChE) as a biomarker for neurological outcomes in TSCI patients. In this retrospective cohort study, 80 cervical TSCI patients (stratified by ASIA impairment scale [AIS]: groups A–D) and 60 healthy controls were analyzed. Serum ChE levels were measured on days 1, 7, and 14 post-injury. Clinical outcomes and correlations between ChE levels and neurological recovery were assessed using analysis of variance, χ² tests, and Spearman’s analysis. ChE levels did not differ across groups at day 1. By days 7 and 14, ChE levels significantly decreased in all TSCI groups compared to controls (*P* < .05). Severe injury groups (AIS A/B) exhibited lower ChE levels than milder groups (AIS C/D) at both timepoints (*P* < .05). Lower baseline ChE correlated with poorer neurological recovery and stronger associations with inflammatory/oxidative stress markers. Serum ChE levels inversely correlate with TSCI severity and predict long-term neurological outcomes, highlighting its potential as a prognostic biomarker.

## 1. Introduction

Traumatic spinal cord injury (TSCI) is among the most catastrophic forms of trauma, disproportionately affecting younger individuals.^[1]^ Pathophysiologically, the primary injury phase is characterized by mechanical disruption of the spinal cord, caused by shearing forces, lacerations, acute stretching, and sudden acceleration-deceleration events.^[2,3]^ This is followed by a secondary injury phase, driven by complex inflammatory cascades involving excitotoxicity, ischemia/hypoxia, inflammatory responses, increased intraparenchymal pressure, and oxidative stress. These mechanisms collectively determine the extent of neuronal loss after the initial mechanical insult.^[4,5]^ The chronic phase is marked by adaptive processes, potential recovery, or the development of autonomic dysregulations.^[3,4,6,7]^ Due to the dynamic and multifaceted nature of these pathophysiological changes, a reliable assessment of preserved or regained neurological function can only be achieved months post-injury, once a new physiological equilibrium is established. Consequently, there is an urgent need for objective, early-stage biomarkers capable of accurately evaluating the extent of damage and providing prognostic insights into potential recovery.^[8]^

Since the early 2000s, the relationship between serum biomarkers and prognostic indicators has gained substantial traction in medical research. Blood-based parameters, due to their accessibility and cost-effectiveness in clinical practice, have emerged as valuable tools for prognostic assessment.^[9]^ Among these, serum enzymes such as lactate dehydrogenase,^[10]^ alkaline phosphatase,^[11]^ γ-glutamyltransferase, and alanine aminotransferase have become key areas of investigation.^[12]^ Within this category, serum cholinesterase (ChE), also referred to as butyrylcholinesterase or pseudocholinesterase, is an alpha-glycoprotein that primarily catalyzes the hydrolysis of acetylcholine and other choline esters.^[13,14]^ Synthesized mainly in the liver and rapidly released into the bloodstream, ChE has been implicated in a variety of pathological conditions. Growing evidence suggests that reduced ChE levels are associated with a range of diseases and clinical states, including chronic liver dysfunction, systemic inflammation, infections, and malnutrition.^[13]^ Recent research has further highlighted the prognostic value of serum ChE in solid tumors. Decreased ChE levels have been correlated with poorer outcomes in several cancers, regardless of hepatic involvement, such as bladder cancer,^[15]^ pancreatic cancer,^[14]^ breast cancer,^[16]^ and colorectal cancer.^[17]^ Notably, a pivotal study by Martinez-Moreno et al has demonstrated the role of ChE in lung cancer biology, emphasizing its significance in oncology research.^[18]^ Collectively, these findings underscore the potential of ChE as a versatile biomarker with prognostic relevance across a spectrum of disease states.

This study investigates the association between ChE levels and disease prognosis in patients with TSCI using a large-scale patient cohort. Our primary objective is to evaluate the prognostic potential of serum ChE as a biomarker for TSCI, focusing on its predictive value for clinical outcomes and its implications for therapeutic decision-making.

## 2. Materials and methods

### 2.1. General information and grouping

This study was conducted in compliance with the ethical standards of the responsible institution on human subjects as well as with the Helsinki Declaration. This retrospective clinical study, approved by the Ethics Committee of the Second People’s Hospital of Changzhou, analyzed 80 patients with traumatic cervical spinal cord injury admitted to the hospital’s Trauma Center between January 2016 and December 2024. All patient data were anonymized and retrospectively analyzed. Informed consent was waived by the ethics committee due to the retrospective design and use of de-identified data. The cohort comprised 67 males (87.5%) and 13 females (12.5%), aged 18 to 89 years (mean ±  standard deviation [SD]: 51.22 ± 15.6 years). Based on the ASIA impairment scale (AIS), patients were stratified into 4 groups: group A (n = 20, 25%), group B (n = 28, 35%), group C (n = 15, 18.7%), and group D (n = 17, 21.3%). A control group of 60 healthy individuals (49 males, 81.6%; 11 females, 19.4%) undergoing routine physical examinations during the same period was included, with an age range of 23 to 62 years (mean ± SD: 50.3 ± 10.2 years). No significant differences in age or gender distribution were observed among the 5 groups (*P* > .05, 1-way analysis of variance).

### 2.2. Inclusion and exclusion criteria

Inclusion criteria: Patients with acute cervical SCI, confirmed by both computed tomography and magnetic resonance imaging within 24 hours post-admission, without concomitant traumatic brain injury or significant injury to other organ systems.

Exclusion criteria: Individuals with a history of prior spinal surgery, preexisting spinal injuries, or neuromuscular disorders were excluded. Additionally, patients presenting with severe systemic comorbidities, including organ dysfunction or failure, or a documented history of malignant neoplasms, were excluded from the study.

### 2.3. ASIA impairment scale

Grade A (complete injury): Absence of both motor and sensory function in sacral segments S4 to S5.

Grade B (sensory incomplete injury): Preservation of sensory function below the neurological level, including sacral segments S4 to S5, with complete absence of motor function.

Grade C (motor incomplete injury): Preservation of motor function below the neurological level, with more than 50% of key muscles demonstrating a muscle grade <3, accompanied by intact sensory function.

Grade D (Motor incomplete injury): Preservation of motor function below the neurological level, with at least 50% of key muscles maintaining a muscle grade ≥3.

Grade E (Normal function): Full preservation of both motor and sensory functions.

### 2.4. Specimen collection

Peripheral venous blood samples (5 mL) were obtained from spinal cord injury patients at 3 time points: days 1, 7, and 14 post-admission. Serum ChE activity in healthy controls was assessed during routine health examinations. All samples were processed in the hospital’s central laboratory using a chemiluminescence-based Roche analyzer.

### 2.5. Statistical analysis

Data analysis was performed using SPSS 26.0 (SPSSInc., Chicago). Continuous variables are presented as mean ±  SD and compared via 1-way analysis of variance for multigroup analyses. Categorical variables are expressed as percentages (%) and analyzed using the χ² test. Correlation analyses were conducted with Spearman’s rank correlation method. Statistical significance was defined as *P* < .05.

## 3. Results

### 3.1. Patients

The study cohort comprised 66 patients in groups A to D, with a male predominance (n = 58, 87.9%) and a median age of 42.5 years (IQR 23–62 years). The primary etiologies of TSCI was traffic (78.8%). The control group, consisting of 60 age-matched individuals (50 males, 10 females; median age 48.8 years). Comparative analysis within the 5 groups (control group, A group, B group, C group, D group) revealed no statistically significant differences in demographic parameters (age, sex), and etiology factor. Detailed demographic and clinical characteristics are presented in Tables 1 and 2.

**Table 1:** General information of the patients.

Term	Age (mean ± SEM)	Sex (n = 60)	Etiology
Control group	48.8 ± 1.19	Male: 50 (83.3%)	
		Female: 10 (16.7%)	
A group	55 ± 2.20	Male: 15	Traffic: 14
		Female: 1	Fall: 2
			Others: 0
B group	48.58 ± 2.52	Male: 21	Traffic:14
		Female: 3	Fall: 2
			Others: 0
C group	51.5 ± 2.91	Male: 10	Traffic: 10
		Female: 2	Fall: 1
			Others: 1
D group	50.29 ± 2.34	Male: 12	Traffic: 14
		Female: 2	Fall: 0
			Others: 0
*P*-value	.2238	.235	.415

SEM = standard error of the mean.

**Table 2:** The American Spinal Injury Association impairment scale (AIS).

AIS grade		Clinical state
A	Complete	There is no preservation of motor or sensory function in the sacral segments S4–S5
B	Incomplete	Sensory but not motor function is preserved below the NLI and includes the sacral segments S4–S5
C	Incomplete	Sensory function is preserved below the NLI, including sacral segments S4–S5, while motor function is not
D	Incomplete	Motor function is preserved below the NLI, with at least half of the key muscles below this level having a muscle grade of 3 or higher
E	Normal	Motor and sensory function is normal

AIS grades A to E are determined based on the completeness of paralysis and the results of motor and sensory function tests.

AIS = ASIA impairment scale, NLI = neurological level of injury.

### 3.2. Biochemical analysis of the serum samples

Serum analysis revealed detectable and quantifiable concentrations of mean arterial pressure, white blood cells, hemoglobin, alanine aminotransferase, albumin, serum creatinine. Notably, there were no statistically significant differences among the 5 groups, as illustrated in Table 3.

**Table 3:** Biochemical analysis of the serum samples.

Term	Mean arterial pressure (mm Hg; mean ± SEM)	While blood cells (×10^9^/L; mean ± SEM)	Hemoglobin (g/L; mean ± SEM)	ALT (U/L; mean ± SEM)	Albumin (g/L; mean ± SEM)	Serum creatinine (μmol/L; mean ± SEM)
Control group	80.9 ± 1.2	11.5 ± 0.7	106.0 ± 2.0	29.0 ± 3.1	35.0 ± 1.4	49.0 ± 2.2
A group	80.5 ± 1.2	11.2 ± 0.8	105.3 ± 2.1	28.5 ± 3.2	35.2 ± 1.5	48.5 ± 2.3
B group	81.3 ± 1.1	12.5 ± 0.9	107.8 ± 2.0	30.2 ± 3.1	34.8 ± 1.4	50.2 ± 2.2
C group	82.0 ± 1.3	11.8 ± 0.7	106.5 ± 1.9	29.8 ± 3.0	35.5 ± 1.6	49.8 ± 2.1
D group	81.8 ± 1.4	12.0 ± 0.8	108.2 ± 2.2	31.0 ± 3.3	34.9 ± 1.5	51.0 ± 2.4
*P*-value	.45	.32	.28	.50	.67	.40

ALT = alanine aminotransferase, SEM = standard error of the mean.

### 3.3. Serum ChE levels demonstrate an inverse correlation with neurological impairment severity

**Figure 1.**
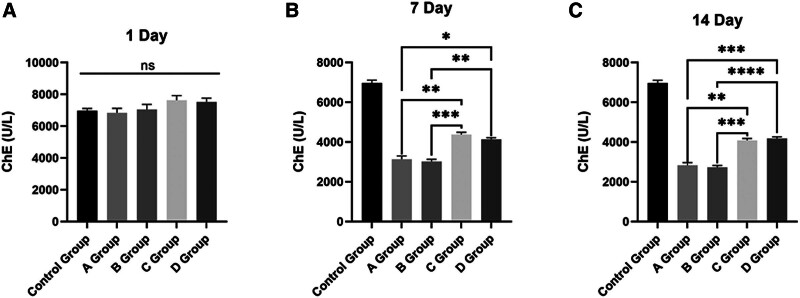
Serum ChE levels demonstrate an inverse correlation with neurological impairment severity. (A) ChE expression among the 5 groups at 1 day. (B) ChE expression among the 5 groups at 7 days. (C) ChE expression among the 5 groups at 14 days. Data are represented as mean ± standard error of mean (mean ± SEM). All *P* values were determined by 1-way ANOVA with Tukey’s post hoc test. *p < .05, **P < .01, ***P < .001, ns, not significant. ANOVA = analysis of variance, ChE = cholinesterase, SEM = standard error of the mean.

As shown in Figure 1A, these 5 groups exhibited no statistical difference in concentration of ChE at 1 day (control group mean ± SEM: 6967 ± 143.3; A group mean ± SEM: 6836 ± 288.0; B group mean ± SEM: 7046 ± 322.4; C group mean ± SEM: 7630 ± 292.0; D group mean ± SEM: 7523 ± 243.7; Fig. 1A).

However, these 5 groups exhibited significant statistical differences in concentration of ChE at 7 day. Compared to the control group, the ChE concentration decreased in groups A to D. Moreover, groups A and B exhibited more severe damage, which corresponded to lower ChE concentrations compared to groups C and D. Additionally, no statistically significant differences were observed between groups A and B or between groups C and D (control group mean ± SEM: 6967 ± 143.3; A group mean ± SEM: 3128 ± 170.3; B group mean ± SEM: 3018 ± 111.4; C group mean ± SEM: 4378 ± 109.9; D group mean ± SEM: 4135 ± 80.57; Fig. 1B).

Furthermore, tremendous pronounced statistical disparities in ChE concentration were observed among the 5 groups at the 14 days. Specifically, groups A through D showed a decrease in ChE concentration relative to the control group. Notably, groups A and B, which exhibited more severe damage, had correspondingly lower ChE concentrations than groups C and D. Furthermore, no statistically significant differences were found between groups A and B or between groups C and D (control group mean ± SEM: 6967 ± 143.3; A group mean ± SEM: 2832 ± 131.0; B group mean ± SEM: 2733 ± 89.07; C group mean ± SEM: 4082 ± 101.2; D group mean ± SEM: 4182 ± 81.32; Fig. 1C).

## 4. Discussion

TSCI represents a devastating form of trauma, disproportionately affecting younger populations and resulting in profound long-term disability.^[19–21]^ The pathophysiology of TSCI encompasses 2 distinct phases: an immediate mechanical insult followed by secondary injury cascades – including neuroinflammation, oxidative stress, and ischemia-reperfusion injury – that collectively drive neuronal degeneration and functional deficits.^[19,22]^ Given the dynamic complexity of these processes, the identification of robust biomarkers capable of early prognostication and therapeutic stratification is critically needed. Here, we investigate ChE as a candidate biomarker for TSCI, evaluating its association with injury severity and clinical outcomes.

Our data demonstrate that serum ChE levels correlate inversely with the severity of neurological impairment in TSCI patients. Specifically, individuals classified as AIS grade A (complete injury) exhibited significantly lower ChE levels compared to those with AIS grade B and D (incomplete injuries). This finding aligns with prior evidence linking ChE depletion to systemic pathologies such as chronic inflammation, hepatic dysfunction, and catabolic states. The observed ChE reduction in TSCI may reflect systemic inflammatory activation and oxidative burden post-injury, positioning ChE as a potential surrogate marker of global injury load.

Notably, ChE levels at admission predicted long-term neurological recovery, with lower baseline values associated with poorer AIS score improvements at follow-up. This prognostic capacity aligns with emerging evidence implicating ChE in modulating neuroinflammation and redox homeostasis – key drivers of secondary injury and repair mechanisms in TSCI. The utility of ChE as an early prognostic tool could inform clinical decision-making, particularly in stratifying patients for targeted therapies.

Further supporting its pathophysiological relevance, ChE levels showed strong correlations with canonical biomarkers of neuroinflammation (e.g., CCL-2, CCL-3) and oxidative stress (e.g., zinc).^[23–25]^ These associations suggest that ChE integrates into a broader biomarker network reflecting multidimensional injury dynamics in TSCI, including immune dysregulation, free radical-mediated damage, and tissue repair processes. The robust linkage between ChE and these markers underscores its potential as a composite indicator of inflammatory and oxidative stress pathways.

## 5. Conclusion

Our study provides compelling evidence that serum ChE is a promising biomarker for TSCI, with potential applications in both the assessment of injury severity and the prediction of long-term outcomes. The strong association between ChE levels and markers of inflammation and oxidative stress further supports its role in the pathophysiology of TSCI. As a readily accessible and cost-effective biomarker, ChE has the potential to enhance the early diagnosis and management of TSCI, ultimately improving patient outcomes. Future research should focus on validating these findings in larger cohorts and exploring the therapeutic implications of modulating ChE levels in TSCI patients.

## Author contributions

**Conceptualization:** Yongchang Li.

**Data curation:** Yongchang Li.

**Formal analysis:** Yongchang Li.

**Investigation:** Yongchang Li.

**Methodology:** Yongchang Li.

**Project administration:** Yongchang Li.

**Resources:** Yongchang Li.

** Software:** Liangyun Shen.

**Supervision:** Liangyun Shen.

**Visualization:** Liangyun Shen, Yongchang Li.

**Writing – review & editing:** Liangyun Shen, Yongchang Li.

**Writing – original draft:** Yongchang Li.
